# Genome of an early Okhotsk individual reveals ancient admixture between Jomon and Kamchatka lineages

**DOI:** 10.1038/s41598-025-21522-4

**Published:** 2025-10-27

**Authors:** Takehiro Sato, Daisuke Kubo, Yu Hirasawa, Minoru Yoneda, Ryosuke Kimura, Atsushi Tajima, Hirofumi Kato

**Affiliations:** 1 Department of Bioinformatics and Genomics, Graduate School of Medical Sciences, Kanazawa University, Kanazawa, 920-8640 Japan; 2 Department of Human Biology and Anatomy, Graduate School of Medicine, University of the Ryukyus, Ginowan, 901-2720 Japan; 3 The Hokkaido University Museum, Hokkaido University, Sapporo, 060-0809 Japan; 4 Department of International Studies, Faculty of Human Sciences, University of East Asia, Shimonoseki, 751-8503 Japan; 5 The University Museum, The University of Tokyo, Tokyo, 113-0033 Japan; 6 Centre for Ainu and Indigenous Studies, Hokkaido University, Sapporo, 060-0808 Japan

**Keywords:** Prehistoric Okhotsk people, Paleogenomics, Next-generation sequencing, Population genetics, Evolution, Genetics

## Abstract

The prehistoric Okhotsk culture was distributed along the southern coastal regions of the Sea of Okhotsk during the late first millennium AD. A previous study that performed whole-genome sequencing of a late Okhotsk individual suggested two migration waves from the Russian Far East to northern Japan. The first wave is estimated to have originated from the Kamchatka Peninsula around 2000 years before present (BP), and the second from the Amur Basin around 1600 BP. These findings suggest the past existence of an admixed hypothetical population between the Kamchatka and indigenous Jomon lineages in northern Japan between 2000 and 1600 BP, although direct genetic evidence has not yet been obtained. Here, we present the genome data of an early Okhotsk individual (NAT004) excavated from northern Japan. Admixture modelling reveals that the genome can be explained as a mixture of Kamchatka and Jomon ancestries, providing direct support for the existence of the previously hypothesized hypothetical population. This result offers new insights into the prehistoric population dynamics of northern Japan and contributes to the broader understanding of its archaeological and anthropological history.

**Supplementary Information:**

The online version contains supplementary material available at 10.1038/s41598-025-21522-4.

## Introduction

**Fig. 1:**
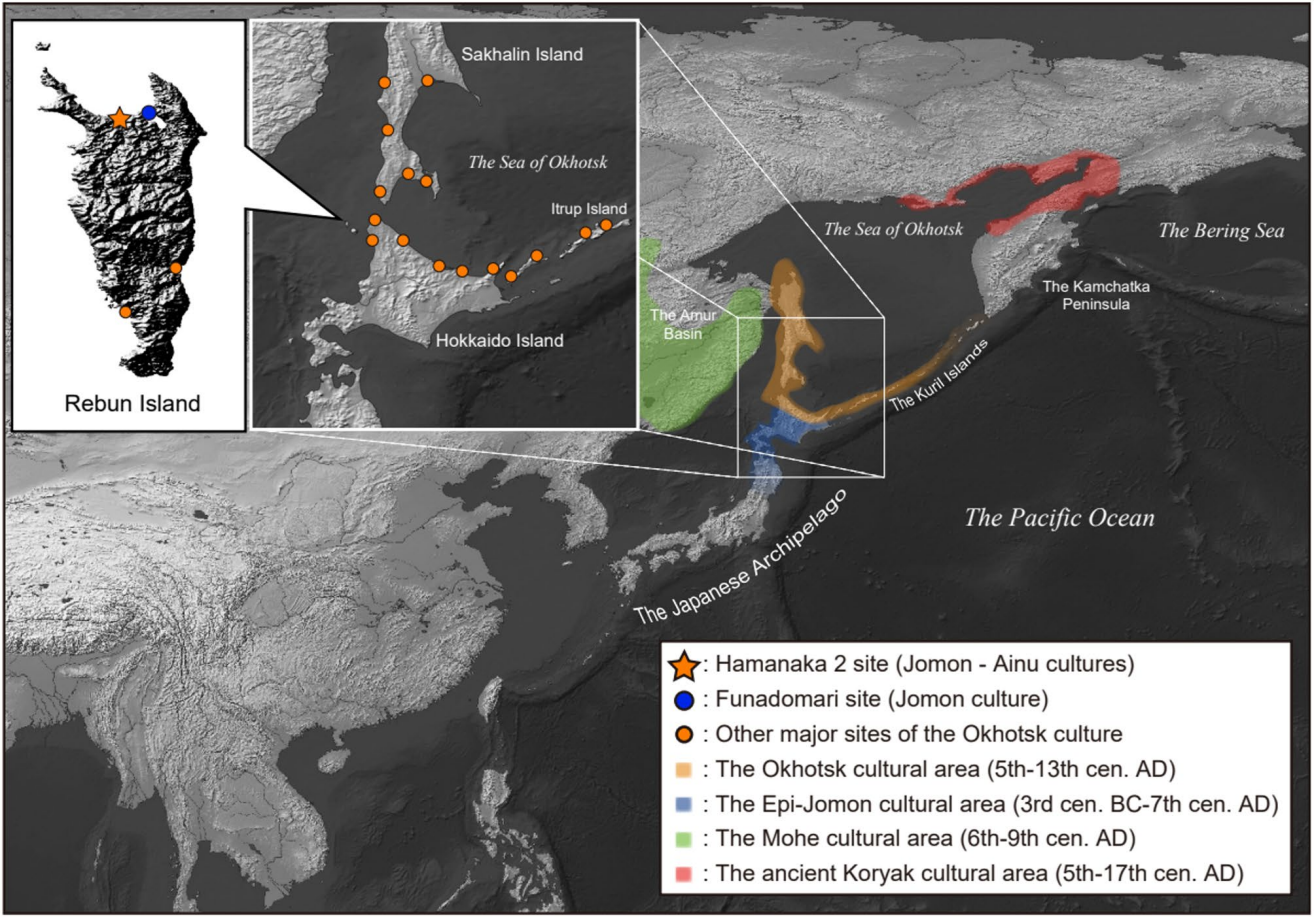
Geographic locations of the Hamanaka 2 site, from which previously reported NAT002^5^ and NAT004 analyzed in the present study were excavated, the Funadomari site from which F23, a previously reported indigenous Jomon individual^6^, was excavated, and other archaeological sites of the Okhotsk culture and geographic ranges of the Okhotsk and other prehistoric cultures around the Sea of Okhotsk. The maps were created with QGIS (https://qgis.org) using raster data downloaded from Natural Earth (https://www.naturalearthdata.com) and ASTER GDEM (https://www.jspacesystems.or.jp).

The Okhotsk culture, a prehistoric marine-adapted hunting and gathering culture, developed around the southern coastal regions of the Sea of Okhotsk, including northern and eastern Hokkaido, Sakhalin, and the Kuril Islands (Fig. 1), from the fifth to the thirteenth centuries AD^1,2^. The distribution of the archaeological sites of the Okhotsk culture is localized in the coastal area, suggesting a dependence on marine resources by the Okhotsk people (Fig. 1), which is supported by zooarchaeological and isotopic studies^3,4^.

Previous studies based on cranial morphology and ancient mitochondrial DNA (mtDNA) have suggested that the Okhotsk people originated in the Lower Amur region^7–10^. Moreover, some of the pottery, iron, and bronze wares excavated from the archaeological sites of the Okhotsk culture are similar to those from sites of the Mohe culture, which developed in the Amur Basin from the 6th to 9th centuries AD^11^. Furthermore, a recent whole-genome analysis of a late Okhotsk individual (NAT002), excavated from the Hamanaka 2 site on Rebun Island in northern Japan (Fig. 1), estimated that approximately 60% of this individual’s ancestry was related to the Amur lineage, indicating that the primary origin of the Okhotsk people was the Amur Basin^5^. In addition to Amur-related ancestry, admixture modelling detected two other ancestry components in the NAT002 genome: Jomon- and Kamchatka-related ancestries. Admixture dating suggests that the mixing of the Jomon- and Kamchatka-related ancestries occurred around 2,000 BP, corresponding to the Epi-Jomon period. This predates the estimated migration from the Amur Basin at approximately 1,600 BP. The Jomon people were the indigenous Neolithic hunter-gatherers of the Japanese archipelago and exhibited unique genetic characteristics in East Asia^12,13^. Given the presence of archaeological sites associated with the Jomon and subsequent Epi-Jomon periods on Rebun Island, it is plausible that people who migrated from the Amur Basin admixed with the indigenous Jomon people. Consequently, the presence of Jomon-related ancestry in the NAT002 genome is consistent with this historical context. However, Kamchatka-related ancestry represents a less anticipated finding for archaeologists. The admixture dating suggests the presence of a hypothetical population that had already formed through mixing between Jomon- and Kamchatka-related ancestries before the arrival of Amur-related ancestries. However, no archaeological evidence for the migration wave from Kamchatka at that time has been discovered to date. The existence of the above hypothetical population is only indirectly inferred from the NAT002 genome, and direct proof of its existence requires the discovery of an individual with Jomon and Kamchatka ancestries. In addition, the previously inferred admixture date of the Amur-related ancestry was assumed to be a single-pulse migration, despite the lack of concrete evidence. Therefore, it remains unclear whether the ancestry proportions of early Okhotsk individuals are similar to those of NAT002.

To address these issues, we conducted a genome analysis of an early Okhotsk individual (NAT004) from the Towada phase, excavated at Hamanaka 2 site (Fig. 1) on Rebun Island, northern Japan. The Towada phase is characterized by pottery featuring tubercle or penetrated circle patterns on the rim, which has been excavated at sites ranging from southern Sakhalin to northern Hokkaido^14,15^. The ancestry of NAT004 can be explained as resulting from the admixture between the Jomon and Kamchatka lineages, with no substantial contribution from the Amur Basin.

## Results and discussion

### Basic statistics and DNA authenticity of NAT004 sequence data

The basic information on the NAT004 genome is summarized in Supplementary Table S1. As a result of shotgun paired-end sequencing and subsequent quality control, forward and reverse reads totalled approximately 2 billion sequence reads obtained from the libraries. Of these, approximately 860 million overlapping forward and reverse reads were merged. The mapping rate of these sequence reads to the reference genome (hs37d5) was 23.3%. After removing duplicate reads, we obtained genome sequence data with an average depth of 0.98× (Supplementary Table S1). The typical deamination pattern characteristic of ancient DNA was observed (Supplementary Figure S1). The modern DNA contamination rates, estimated based on mtDNA using Schmutzi^16^ and on autosomal DNA using hapCon_ROH^17^, were 0.02 (95% CI: 0.01–0.03; Supplementary Figure S2) and 0.00 (95% CI:0.00–5.7 × 10⁻⁵), respectively (Supplementary Table S1, the lower CI bound was truncated at 0, as contamination rates cannot take negative values). The estimated endogenous mtDNA haplotype of NAT004 was assigned to haplogroup D4m2a by HaploGrep 2.0^18^, which is predominantly observed in the Nivkh among the modern human populations^19^. A total of 172 ROH blocks were detected, with a cumulative length of 1744.93 cM, suggesting strong inbreeding in this individual. This is plausible given that Rebun Island is a relatively small landmass geographically separated from Hokkaido Island (Fig. 1), which may have limited the size of the local mating pool during prehistoric periods. The *R*_Y_ value (ratio of reads mapped to Y and X chromosomes), calculated using ry_compute.py^20^ was 0.001 (95% CI: 0.001–0.0011), indicating that NAT004 was female (Supplementary Table S1). This result is consistent with the morphological observations. Collectively, these results support the authenticity of the NAT004 sequence data.

## Population genetic analyses

**Fig. 2:**
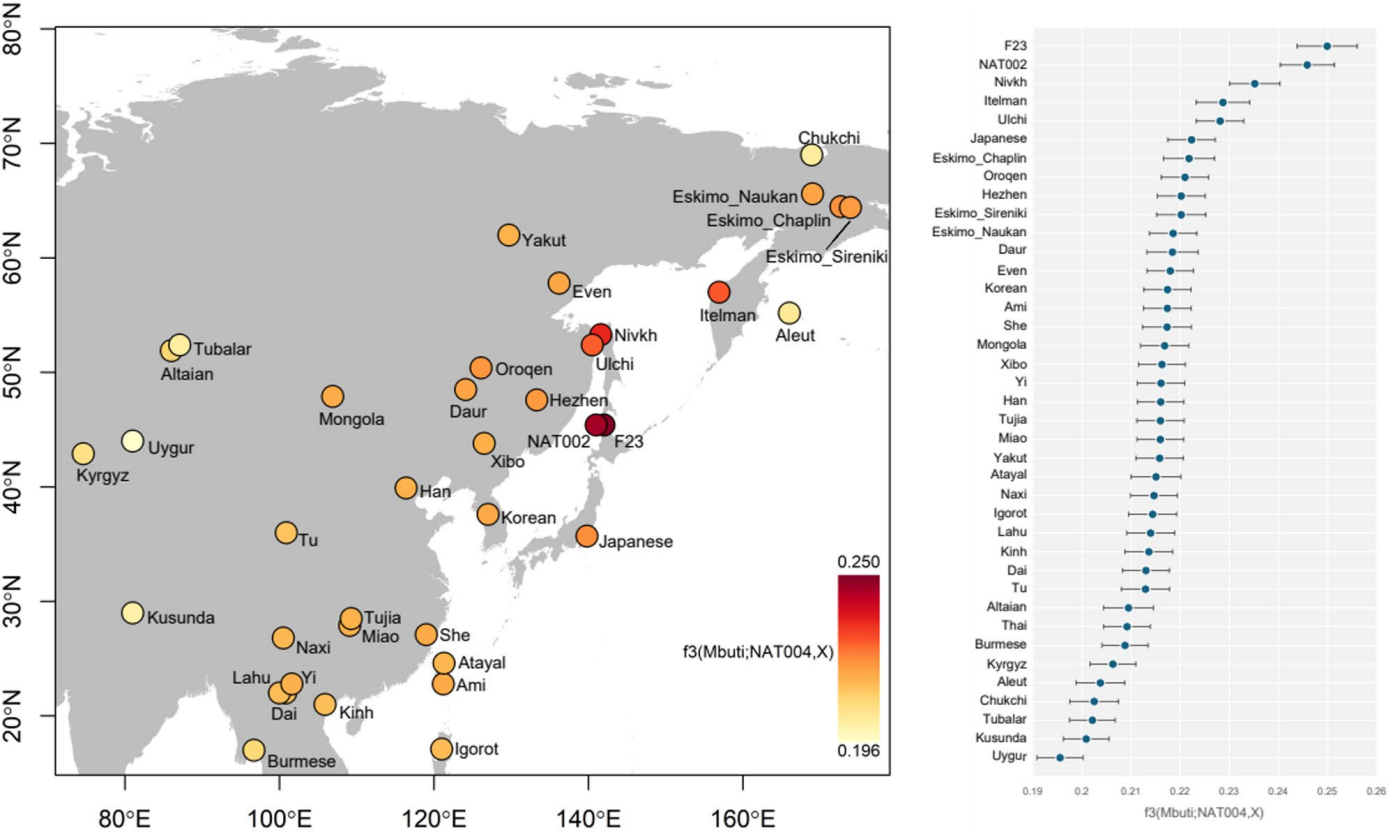
The results of the outgroup *f*_3_ test. *f*_3_(Mnuti; NAT004, X) was calculated (modern Asian populations, NAT002, and F23 were used as population X). Error bars indicate 2 standard errors.

**Fig. 3:**
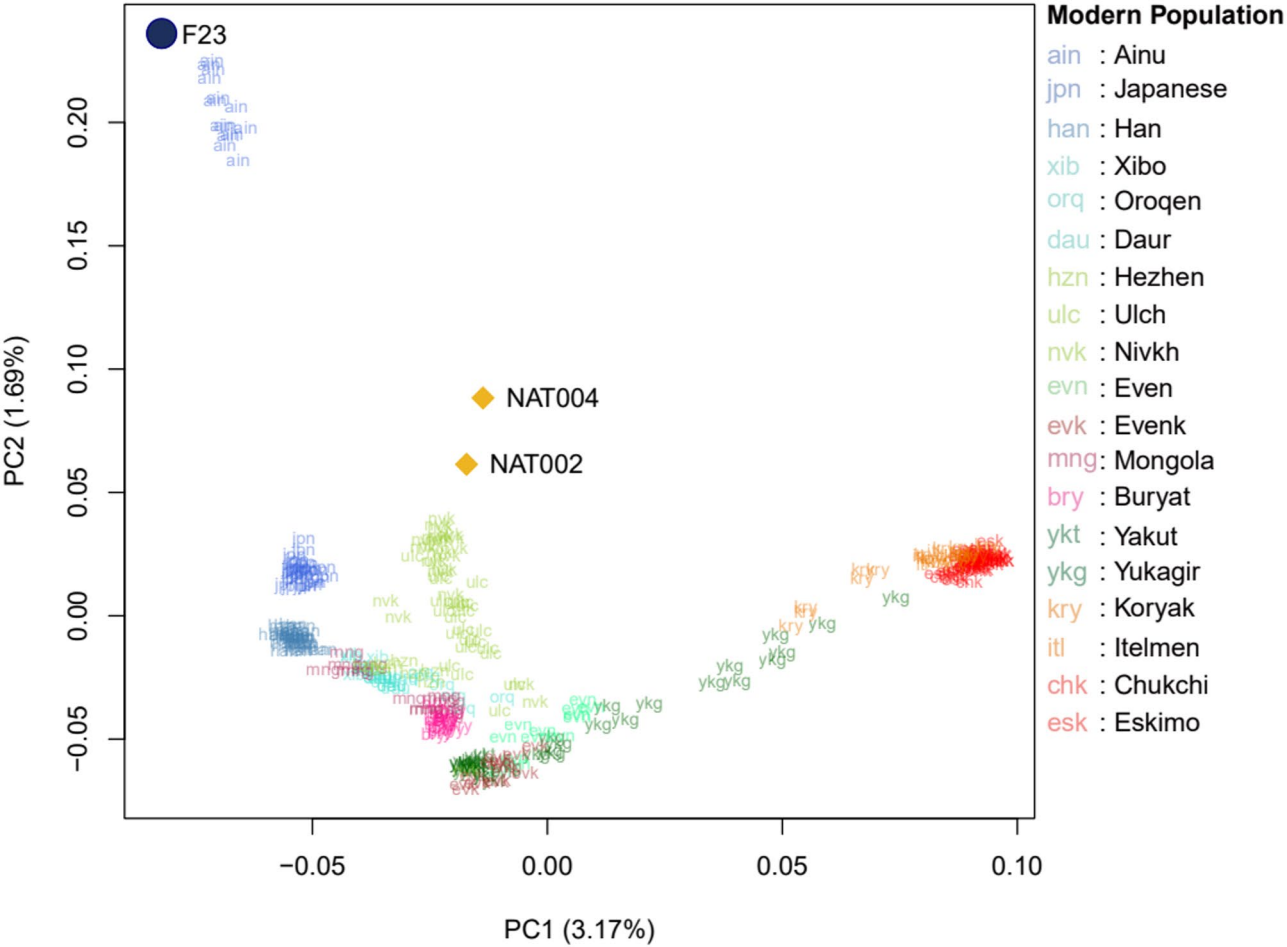
PCA plot of East and Northeast Asian populations based on all SNV sites. NAT004 was projected onto the PC1-PC2 surface calculated based on the genotypes of modern East and Northeast Asian populations and two high-coverage ancient genomes (NAT002 and F23).

The outgroup *f*_3_-test indicated that NAT004, an early Okhotsk individual, had strong genetic affinities, especially with the late Jomon (F23)^6^, late Okhotsk (NAT002)^5^, and Nivkh, Itelman, and Ulchi individuals (Fig. 2). Unexpectedly, the individual showing the strongest genetic affinity to NAT004 was F23, a Jomon individual (*f*_3_(Mbuti; NAT004, F23) = 0.2499 ± 0.0031), rather than NAT002, an individual from the same Okhotsk culture as NAT004 (*f*_3_(Mbuti; NAT004, NAT002) = 0.2459 ± 0.0028). In the PCA plot based on principal components calculated from modern populations and two high-coverage ancient genomes (F23 and NAT002; Fig. 3), NAT004 is positioned closer to NAT002 than to F23, suggesting shared genetic characteristics with NAT002, although subtle differences remain. In particular, NAT004 shows a slightly higher PC2 score than NAT002. This pattern may imply that NAT004 possesses somewhat less Amur-related ancestry than NAT002. Similar patterns were also observed when the analyses were restricted to transversion sites (Supplementary Table S2 and Supplementary Figure S3). Spearman’s ρ between *f*_3_(Mbuti; NAT004, X) values based on all SNV sites and transversion sites was extremely high (ρ = 0.997). In addition, F23, NAT002, and NAT004 were projected onto a PCA plot based on principal components from modern populations, with NAT002 and NAT004 maintaining a consistent relative position (Supplementary Figure S4).

**Fig. 4:**
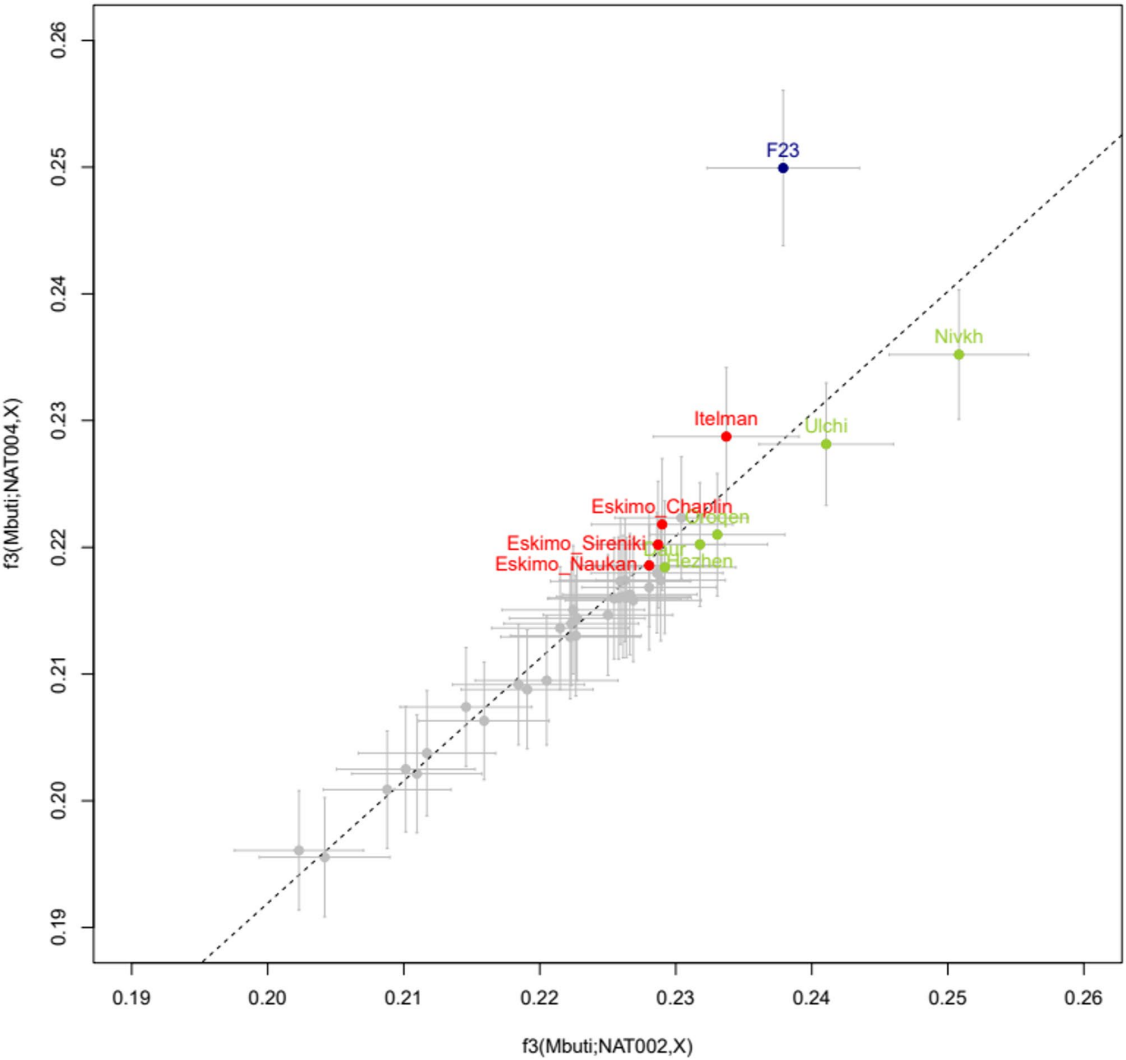
The pairwise *f*_3_ test. The horizontal axis and vertical axis indicate *f*_3_(Mbuti; NAT002, X) and *f*_3_(Mbuti; NAT004, X), respectively. Error bars indicate 2 standard errors. The dashed line indicates the regression line.

**Fig. 5:**
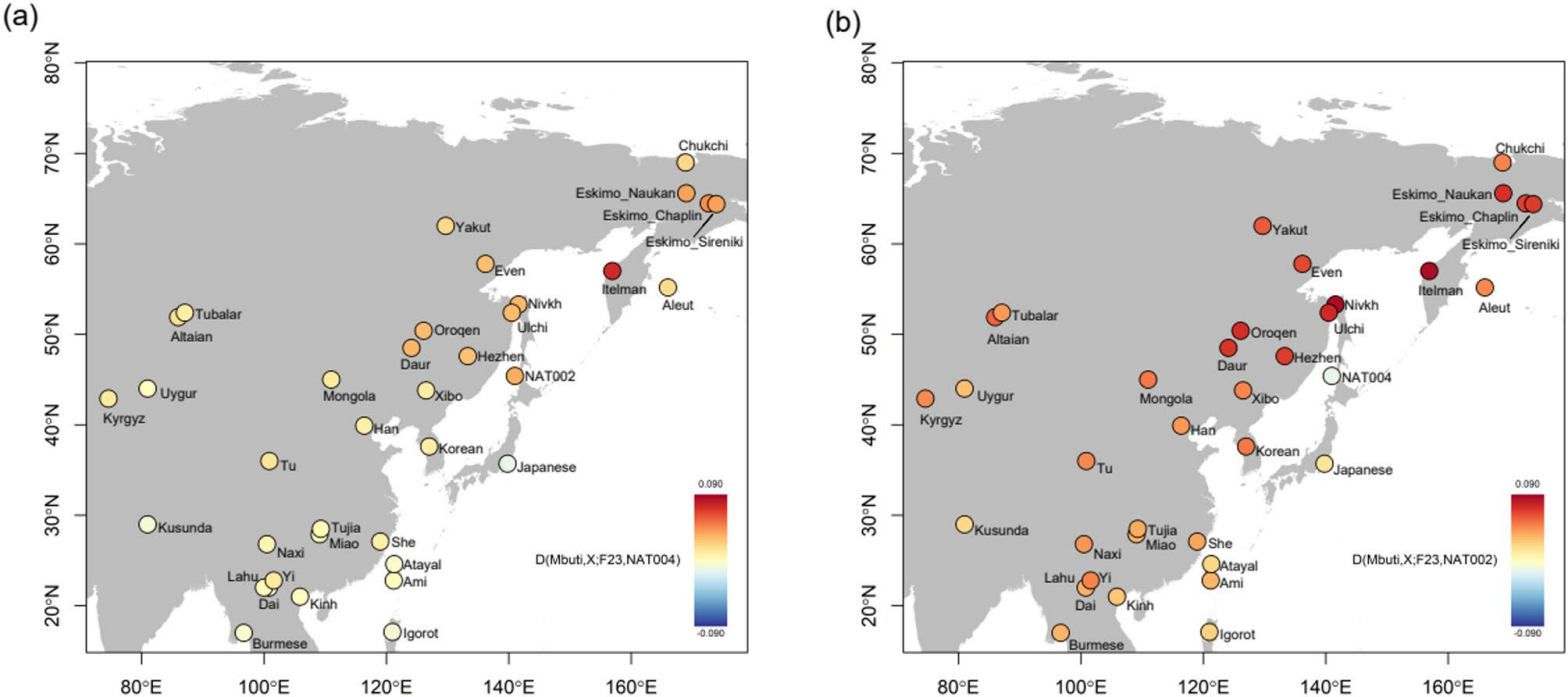
**a** *D*(Mbuti, X; F23, NAT004). The result indicates that NAT004, the early Okhotsk individual, was strongly affected by the Itelman population but not so strongly affected by the Amur populations. **b** *D*(Mbuti, X; F23, NAT002). The result indicates that NAT002, the late Okhotsk individual, was strongly affected by both the Itelman and the Amur populations.

To explore the populations that contributed to the genetic differences between NAT002 and NAT004, we calculated *D*(Mbuti, X; NAT002, NAT004) (Supplementary Figure S5). Among the tested populations, only F23 showed a significantly positive value (Z = 5.28), suggesting that F23 is more closely related to NAT004 than to NAT002. In contrast, all tested populations, except for Itelman and F23, showed significant negative values (Z < -3.0). Similar patterns were observed when the analysis was restricted to transversion sites (Supplementary Table S3). Spearman’s ρ between *D*(Mbuti, X; NAT002, NAT004) values based on all SNV sites and transversion sites was extremely high (ρ = 0.982). These *D* test results appear to be strongly biased, likely due not only to postmortem deamination but also, and more importantly, to reference bias in the low-coverage NAT004 genome. Therefore, we instead employed pairwise outgroup *f*_3_ tests, which are less sensitive to this bias. In the pairwise *f*_3_ plot for *f*_3_(Mbuti; NAT002, X) vs. *f*_3_(Mbuti; NAT004, X) (Fig. 4), Amur populations—including the Nivkh, Ulch, Oroqen, Daur, and Hezhen—tend to plot below the regression line. In contrast, F23 and Itelman tend to plot above the regression line. These findings suggest that the Amur populations are more closely related to NAT002 than to NAT004, whereas F23 and Itelman are more closely related to NAT004 than to NAT002. However, aside from the Nivkh and F23, none of the other populations exhibit statistically significant deviations from the regression line. Moreover, we calculated *D*(Mbuti, X; F23, NAT004) and *D*(Mbuti, X; F23, NAT002) to evaluate genetic changes from the late Jomon to early or late Okhotsk periods (Fig. 5). *D* statistics indicated that NAT004 was strongly affected by the Itelman population but not so strongly affected by the Amur populations (Fig. 5a), whereas NAT002 was strongly affected by both the Itelman and Amur populations (Fig. 5b). Although *D*(Mbuti, X; F23, NAT004) might also have been affected by reference bias due to the low-coverage NAT004 genome, the results suggest that NAT004 was strongly affected by Itelman compared to F23. In this case the reference bias of the low-coverage NAT004 genome must have led the *D* statistics in a negative direction because part of the human reference genome is apparently derived from Africans; therefore, positive *D* statistics should not be overestimated. The pairwise *f*_3_ tests yielded similar results. In the plot of *f*_3_(Mbuti; F23, X) vs. *f*_3_(Mbuti; NAT004, X), the Itelman, Eskimo_Chaplin, Eskimo_Naukan, and Eskimo_Sireniki populations significantly deviated upward from the regression line, suggesting that the Kamchatka/Chukotka population genetically affected the population in northern Japan from the late Jomon to early Okhotsk periods, whereas the Amur populations, such as the Nivkh, Ulch, Oroqen, Hezhen, and Daur, did not significantly deviate from the regression line (Supplementary Figure S6a). In the plot of *f*_3_(Mbuti; F23, X) vs. *f*_3_(Mbuti; NAT002, X), both the Kamchatka/Chukotka and Amur populations deviated significantly from the regression line (Supplementary Figure S6b). The admixture signals of the Amur populations, such as the Nivkh, Ulch, Oroqen, Hezhen, and Daur populations, in both the pairwise *f*_3_ and *D* tests were weaker than those of the Kamchatka/Chukotka populations in the NAT004 genome (Fig. 5a and Supplementary Figure S6a). These genomic features of NAT004 appear to be clearly different from those of NAT002, which was inferred to be strongly influenced by Amur populations rather than by Kamchatka/Chukotka populations (Fig. 5b and Supplementary Figure S6b).

**Fig. 6:**
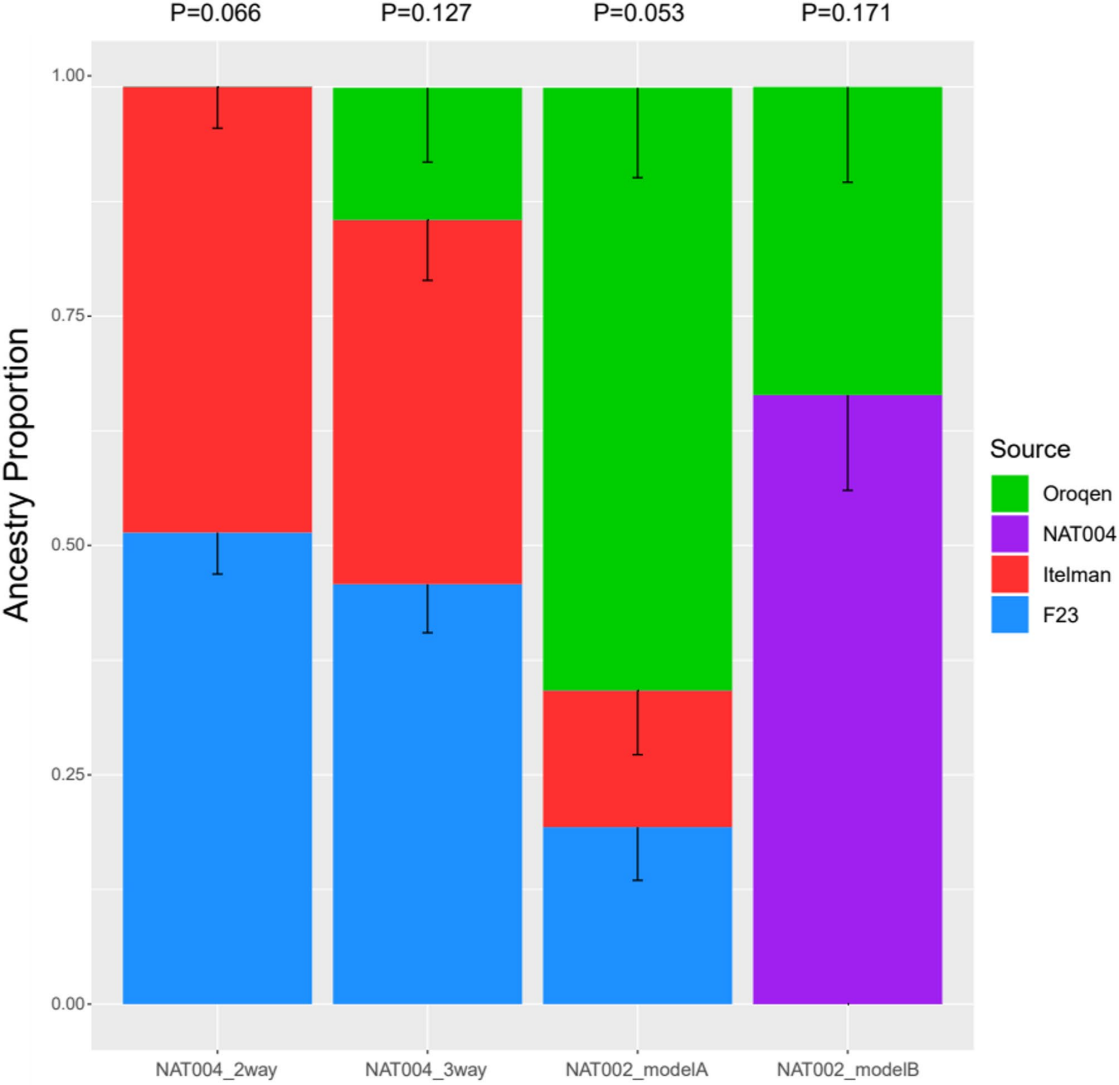
Admixture modelling for NAT002 and NAT004 using qpAdm. The Ami, Dai, Aleut, Mixe, Yakut, and Papuan were used as right populations. F23, Itelman, and Oroqen were used as source populations. In addition, NAT004 was also used as the source population instead of F23 and the Itelman in NAT002_modelB. Error bars indicate 1 standard error.

To assess the genetic changes in the population in this region from the early to the late Okhotsk period in more detail, we performed admixture modelling for NAT004 and NAT002 (Fig. 6). As in a previous study^5^, NAT002 was adequately explained as an admixed individual between the Jomon- (represented by F23), Kamchatka- (represented by Itelman), and Amur-related (represented by Oroqen) ancestries (*P* = 0.0529). In contrast, NAT004 was adequately explained as an admixed individual between the Jomon- and Kamchatka-related ancestries (*P* = 0.0665) without Amur-related ancestry. Although the three-way (Jomon–Kamchatka–Amur) admixture model was also acceptable for NAT004 (*P* = 0.127), the fit to the observed data was not significantly improved compared to the two-way (Jomon-Kamchatka) admixture model (*P* value for nested model = 0.079). When qpAdm modeling was conducted using only transversion sites, both the three-way and two-way models were rejected (*P* = 0.018 and 0.014, respectively), likely owing to the increased variance resulting from the reduced number of variants. Nevertheless, the estimated ancestry proportions remained largely stable, and the conclusion that the three-way model did not provide a significantly better fit than the two-way model remained unchanged (*P* value for the nested model = 0.124; Supplementary Table S4). To confirm that the difference between the admixture modelling results for NAT002 and NAT004 was not due to the difference in genome coverage, we downsampled the NAT002 sequence data to generate 100 datasets with coverage comparable to that of NAT004 (0.98×) and performed admixture modelling on each downsampled dataset (Supplementary Figure S7). None of the 100 downsampled NAT002 datasets were adequately explained by any two-way admixture model (*P* < 0.05). In contrast, 83 datasets were adequately explained by the three-way admixture model (*P* ≥ 0.05), while the remaining 17 were not (*P* < 0.05). This number of poorly fitting datasets exceeds the expected number of false positives due to multiple testing. The excess is likely attributable to loss of precision and sampling variance due to downsampling. Nonetheless, likelihood ratio tests for nested models indicated that the three-way admixture model provided a significantly better fit for all 100 down-sampled NAT002 datasets compared to any two-way admixture model (*P* < 0.05). These findings suggest that the admixture modelling result for NAT004 is not an artifact of low-coverage. Rather, they imply that the proportion of Amur-related ancestry in the NAT004 genome was either zero or significantly lower than that in NAT002. In the three-way admixture model, the Amur ancestry proportion was estimated at 14.4% in NAT004 and 65.7% in NAT002 (Fig. 6 and Supplementary Figure S7).

The ^14^C age of NAT004 was cal AD 409–600 (95.4%), which is extremely close to the previously estimated admixture date (approximately 1,600 BP) of the Amur-related ancestry in this region^5^. Therefore, NAT004 may reflect the genetic characteristics of the population that existed around northern Japan prior to the genetic influence of the migration wave from the Amur Basin. Furthermore, the genetic profile of NAT004, which can be adequately explained as an admixed individual with Jomon- and Kamchatka-related ancestries, is consistent with that of the hypothetical population prior to the migration wave from the Amur Basin, previously inferred using the NAT002 genome^5^. In fact, the NAT002 genome was also adequately explained as an admixed individual between NAT004- and Amur-related ancestries (*P* = 0.171; Fig. 5) but was not explained by NAT004-related ancestry alone (*P* = 1.62 × 10^− 5^ and P value for the nested model was 0.0002). In addition, the NAT004 genome was not adequately explained by Jomon-related ancestry alone (*P* = 1.56 × 10^− 19^ and the *P* value for the likelihood ratio test for the nested model was 4.0 × 10^− 21^). However, the proportion of NAT004-related ancestry (66.4%) in NAT002_modelB was much higher than the sum of the proportions of Jomon- and Kamchatka-related ancestry (34.2%) in NAT002_modelA (Fig. 5). This result implies that the NAT004 genome retains Amur-related ancestry. Indeed, the three-way admixture model for NAT004 (45.8% of the Jomon-, 39.7% of the Kamchatka-, and 14.4% of the Amur-related ancestries) was also acceptable (*P* = 0.127), although this model was not significantly better than the two-way admixture model (51,4% of the Jomon- and 48.6% of the Kamchatka-related ancestries; *P* value for the likelihood ratio test for the nested model was 0.0792). In addition, it is possible that the lack or low proportion of Amur-related ancestry observed in NAT004 may reflect incomplete genetic homogenization within the Okhotsk population during the early Okhotsk period, suggesting that other contemporaneous individuals could have harboured higher proportions of Amur-related ancestry. Even if these possibilities are true, it would still be difficult to explain the existence of individuals with genetic characteristics, such as NAT004, unless a population composed of Jomon- and Kamchatka-related ancestries existed prior to migration from the Amur Basin. Overall, the results obtained in the present study are consistent with the sequence of migration waves inferred in the previous study:^5^ first, the admixture between the Jomon- and Kamchatka-related ancestries occurred, followed by the admixture with the Amur-related ancestry. However, admixture dating estimated that the admixture between Jomon- and Kamchatka-related ancestries occurred 25 ± 8 generations before NAT004, corresponding to 2,250 ± 240 BP, assuming a generation time of 30 years (Supplementary Figure S8). When only transversion sites were used, the admixture event was estimated to have occurred 31 ± 8 generations ago, showing no significant difference from the estimate obtained using all SNV sites. These estimates are slightly older than the previously reported admixture date of 1,950 BP based on the NAT002 genome^5^, although the difference between the estimate for NAT004 using all SNV sites and that for NAT002 is not statistically significant. All three estimates—the two based on the NAT004 genome (using all SNV sites and only transversion sites) and the one based on the NAT002 genome^5^—suggest that the admixture between the Jomon and Kamchatka lineages occurred during the late Jomon and the early Epi-Jomon periods.

One of the limitations of this study is its reliance on genome data derived from only one early (NAT004) and only one late Okhotsk individual (NAT002), assuming that these individuals are representative of the early or late Okhotsk people. However, we cannot completely exclude the possibility that these individuals are migrant individuals from another region (i.e., population outliers). To resolve this issue, further genomic studies using a number of Okhotsk specimens are necessary.

## Conclusion

The NAT004 genome provides direct evidence supporting the existence of a hypothetical population composed of Jomon- and Kamchatka-related ancestries, which was previously inferred from the NAT002 genome. Future analyses targeting a larger number of ancient individuals excavated from northern Japan may more precisely determine the dates and types (single pulse, multiple pulses, or continuous) of migrations from the Kamchatka Peninsula/Amur Basin and the genetic characteristics of the populations before and after the migration events.

## Materials and methods

### Sampling, DNA extraction, library preparation, and sequencing

NAT004 was recovered from the layer corresponding to the early Okhotsk period (Towada phase) at the Hamanaka 2 site (Fig. 1). A bifacial siliceous shale point characteristic of the Towada phase, two impaling tools made of marine animal bone, and three bone needles, thought to be burial items, were excavated from the skeletal remains of NAT004^21^. The discovery of human remains from this period is extremely rare and represents the second case in Japan.

We consulted with the Ainu Association of Hokkaido regarding this research, and the whole-genome analysis was approved by the ethics committee of Kanazawa University. At the excavation site, the first author (T.S.) dug up and sampled the right petrous bone using standard contamination precautions (e.g., wearing latex gloves and face masks). After excavation, the sampled bone was immediately placed in a plastic bag, stored at -30℃, and sent directly to an ancient DNA dedicated laboratory in old campus of Graduate School of Medicine, University of the Ryukyus (207 Uehara, Nishihara, 903 − 0215, Japan). Genomic DNA was extracted from the petrous bone according to the method described by Gamba et al. (2016)^22^. End-repair and adapter ligation steps were performed using the QIAseq Ultralow Input Library Kit (Qiagen) and TruSeq adapters (Illumina) without Uracil-DNA Glycosylase (UDG) treatment. Polymerase chain reaction (PCR) amplification was performed for a reaction mixture of 50 µL containing 5 µL of the genomic DNA library, 25 mL of 2× KAPA HiFi HotStart Uracil + ReadyMix (KAPA Biosystems), 5 mL of primer cocktail in the TruSeq DNA Nano Library Prep Kit (Illumina), and 15 µL of distilled water under the following conditions: 98 °C for 45 s, 12 cycles at 98 °C for 15 s, 60 °C for 3 min, and 72 °C for 30 s, with a final extension at 72 °C for 1 min. PCR products were purified using a Kleen Spin Column (Bio-Rad). Five libraries were prepared, and shotgun sequencing (150 bp PE) was performed using HiSeq X. All pre-PCR experiments were conducted with standard contamination precautions, such as wearing disposable coveralls, gloves, and masks, on a clean bench with positive-pressure air filtering, which was installed in a dedicated experimental room for ancient DNA.

### Mapping and pseudo-haplotype call

Adapter sequences were trimmed from the raw sequence reads using AdapterRemoval v. 2.2.0^23^. We used PRINSEQ-lite^24^ for the quality control procedure under the following conditions: bases with a Phred score < 30 were trimmed from the 5’ and 3’ ends, the sequence reads shorter than 35 bp were removed, and the sequence reads with an average Phred score < 30 were excluded. Sequence reads that passed QC were mapped to the human reference genome (hs37d5) using BWA-MEM^25^. The clipped reads and reads with a mapping quality < 30 were removed from the alignment file (BAM file) using SAMtools^26^. Duplicate reads were excluded using Picard MarkDuplicates (http://broadinstitute.github.io/picard). After indel realignment using the GATK IndelRealigner^27^, the average depth was calculated using the GATK DepthOfCoverage. The Phred score for each base was rescaled based on the damage level using mapDamage 2.0^28^.

Pseudo-haplotype calls were performed using the PileupCaller in SequenceTools (https://github.com/stschiff/sequenceTools) with --randomHaploid option. The resulting pseudo-haplotype data for NAT004 were merged with previously published genome data from modern and ancient Northeast Asians for subsequent population genetic analyses.

## DNA authenticity

To evaluate the authenticity of the NAT004 sequence, we confirmed the deamination pattern using mapDamage 2.0^28^. The mtDNA-based modern DNA contamination rate was estimated using Schmutzi^16^ after re-aligning the mapped reads to the revised Cambridge Reference Sequence, NC_012920.1^29^. The estimated endogenous and contaminant mtDNA haplotypes were assigned to haplogroups using HaploGrep 2^18^. The autosomal DNA-based contamination rate was estimated using hapCon_ROH^17^. The *R*_Y_ value, which is the ratio of reads mapped to Y and X chromosomes, was calculated using ry_compute.py^20^.

## Population genetic analyses

For the population genetic analyses, we prepared two distinct datasets consisting of different populations and single-nucleotide variant (SNV) sets. Dataset 1 consisted of overlapping single-nucleotide polymorphisms (SNPs, generally defined as SNVs with a minor allele frequency of 1% or greater) from previously reported DNA microarray datasets for modern Northeast Asian populations^30–33^ and three ancient genomes from northern Japan: F23^6^, NAT002^5^, and NAT004. This dataset included the genotypes of 49,079 SNPs in 343 Northeast Asian individuals and was used for principal component analysis (PCA). Dataset 2 consisted of 31,201,328 SNVs across 286 previously published modern and ancient Homo sapiens individuals, including high-coverage genomes from the human samples of the Simons Genome Diversity Project (SGDP)^34^ (excluding Neanderthal and Denisovan), two modern Nivkh individuals^35^, two previously published genomes from northern Japan (F23^6^ and NAT002^5^, and NAT004. This dataset was used for the outgroup *f*_3_, *D*, qpAdm modelling, and admixture dating because of the dense SNV data but was not suitable for PCA due to the small number of individuals per population compared to Dataset 1.

The PCA was performed using smartpca in the EIGENSOFT package^36^. Principal components were calculated from modern East and Northeast Asian populations and two high-coverage ancient individuals (F23 and NAT002), and NAT004 was projected onto the PC1-PC2 surface using the lsqproject: YES option. Outgroup *f*_3_ and *D*-tests and qpAdm modelling were performed using qp3Pop, qpDstat, and qpAdm in ADMIXTOOLS^37^, respectively. In the qpAdm model, Ami, Dai, Aleut, Mixe, Yakut, and Papuan were used as the right populations, and NAT002 and NAT004 were modelled using a two-way admixture (F23–Itelman) or three-way admixture (F23–Itelman–Oroqen). Admixture dating for NAT004 was performed using DATES^38^, with F23 and Itelman assumed as source populations.

## Supplementary Information

Below is the link to the electronic supplementary material.

Supplementary Material 1

Supplementary Material 2

## Data Availability

The datasets analysed during the current study are available in the DDBJ Sequence Read Archive (DRA) repository (https://www.ddbj.nig.ac.jp/dra/index-e.html), under the accession numbers PRJDB20690 for the NAT004 genome, newly generated in the present study, and PRJDB8675 for the NAT002 genome, which was previously generated in Sato et al. (2021).
