# Supplementary material for: Genome of an early Okhotsk individual reveals ancient admixture between Jomon and Kamchatka lineages
Source: Sci Rep. 2025 Oct 27;15:37520. doi: 10.1038/s41598-025-21522-4 (PMC12559309; doi:10.1038/s41598-025-21522-4)
Supplement: Supplementary file 2 — Supplementary Material 2 [file 41598_2025_21522_MOESM2_ESM.pdf]

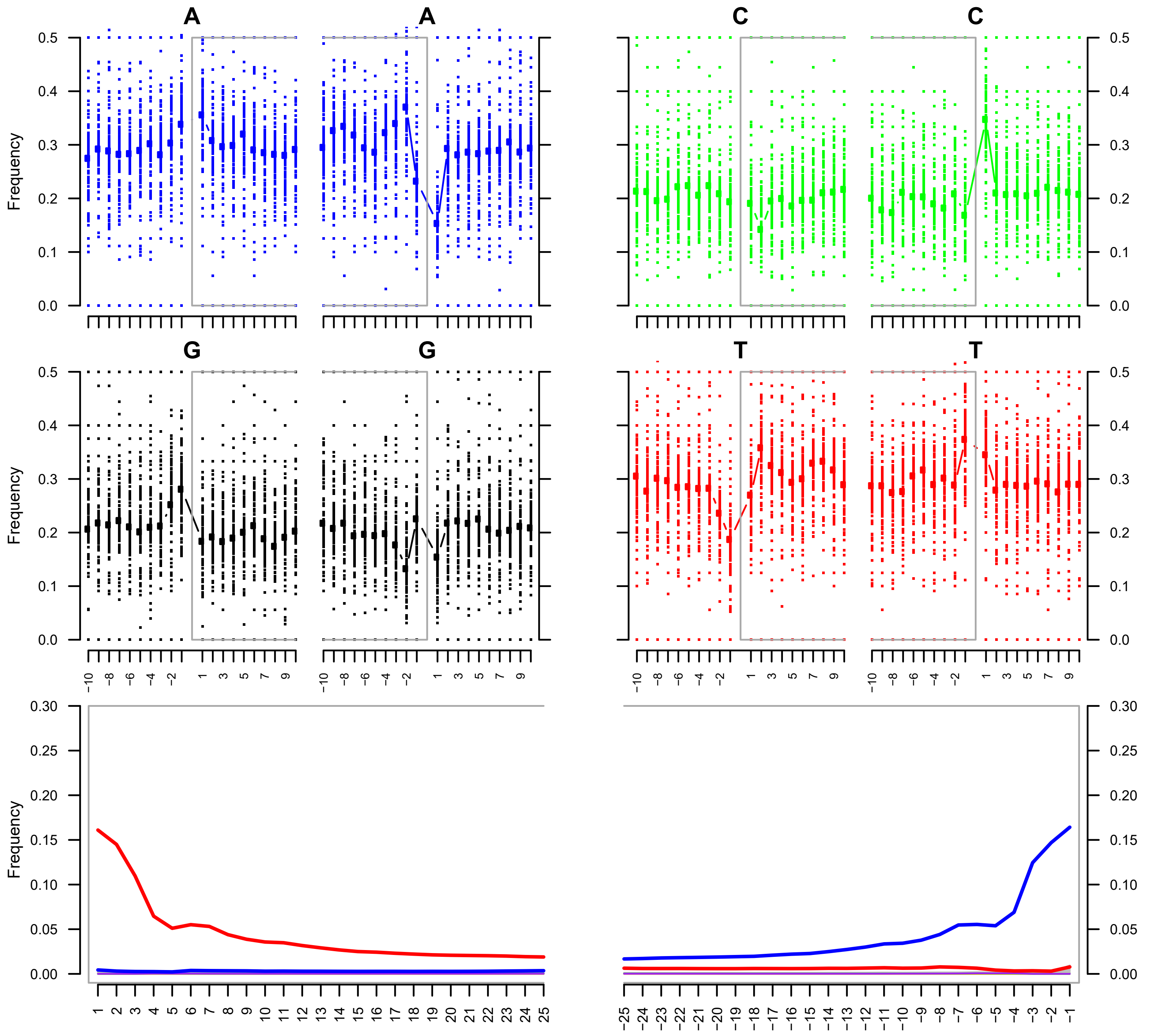


**Supplementary Figure S1** Depurination and deamination patterns observed in NAT004 sequence data. The red and blue lines in the bottom plot indicate C to T and G to A transitions, respectively.

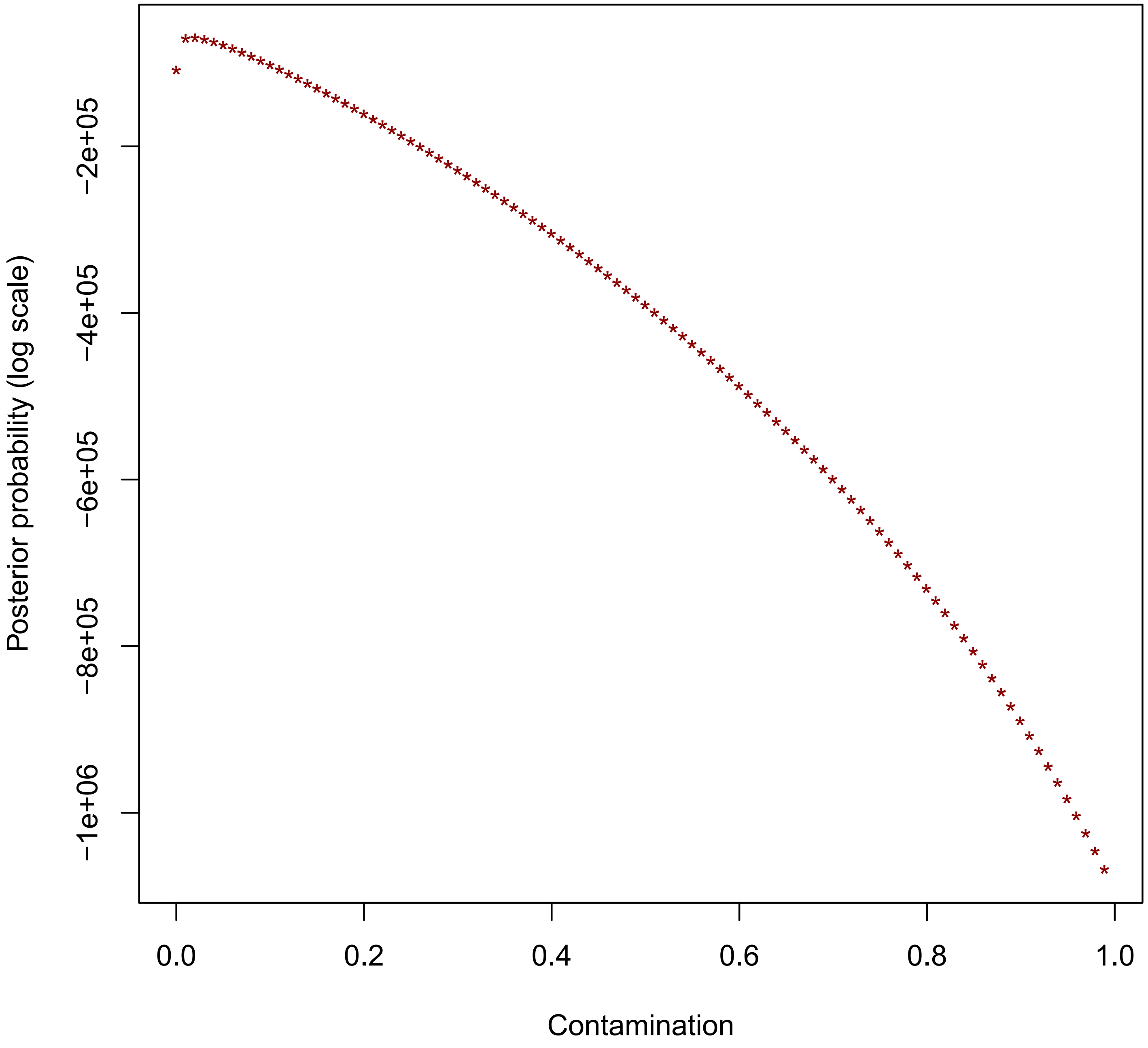


**Supplementary Figure S2** Posterior probability for the modern DNA contamination rate inferred from NAT004 mtDNA sequence data.

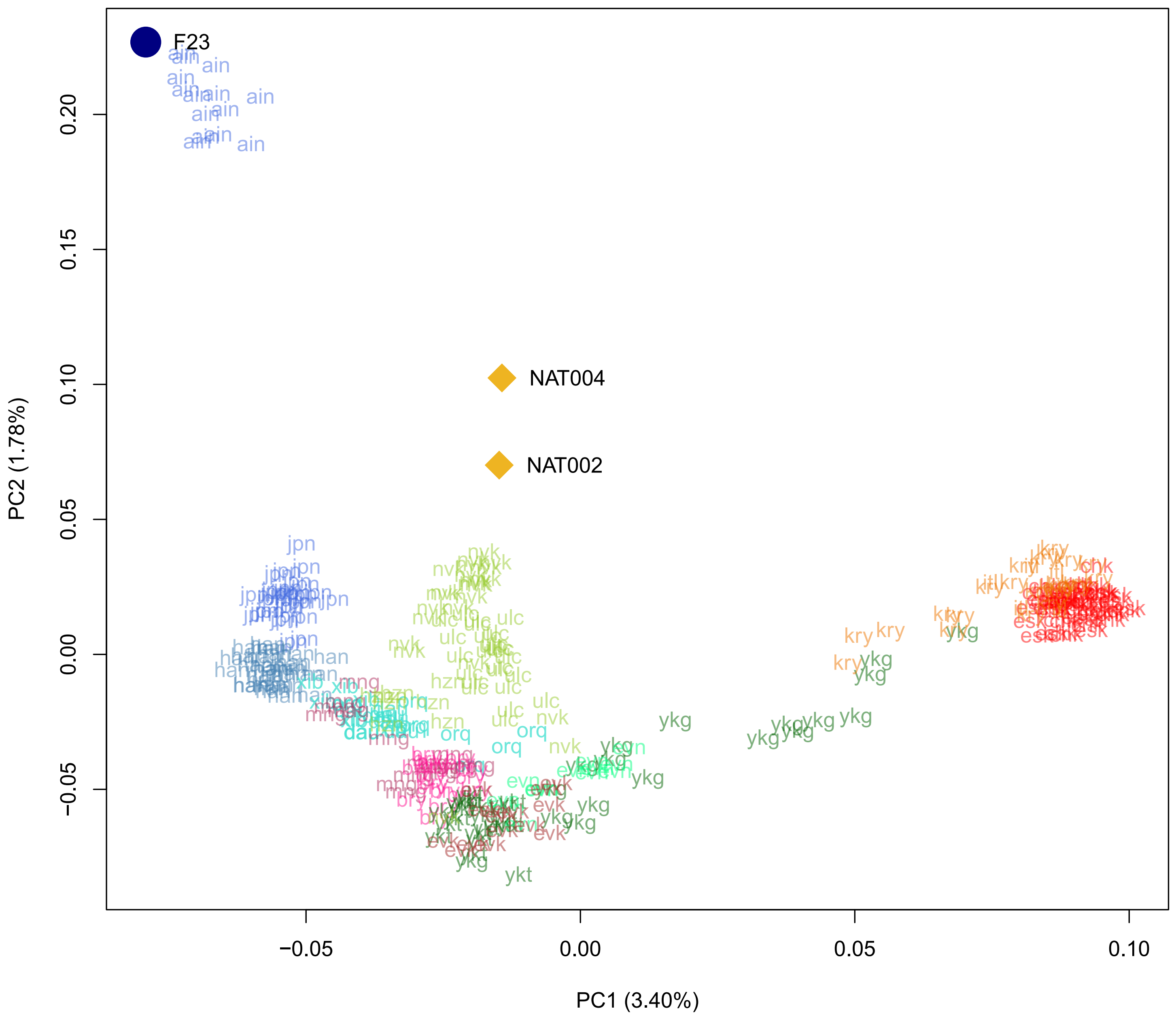


**Supplementary Figure S3** A PCA plot based on transversion  sites only. Principal components were calculated using modern populations and two high-coverage ancient individuals (F23 and NAT002), and NAT004 was projected onto the PC1-PC2 surface.

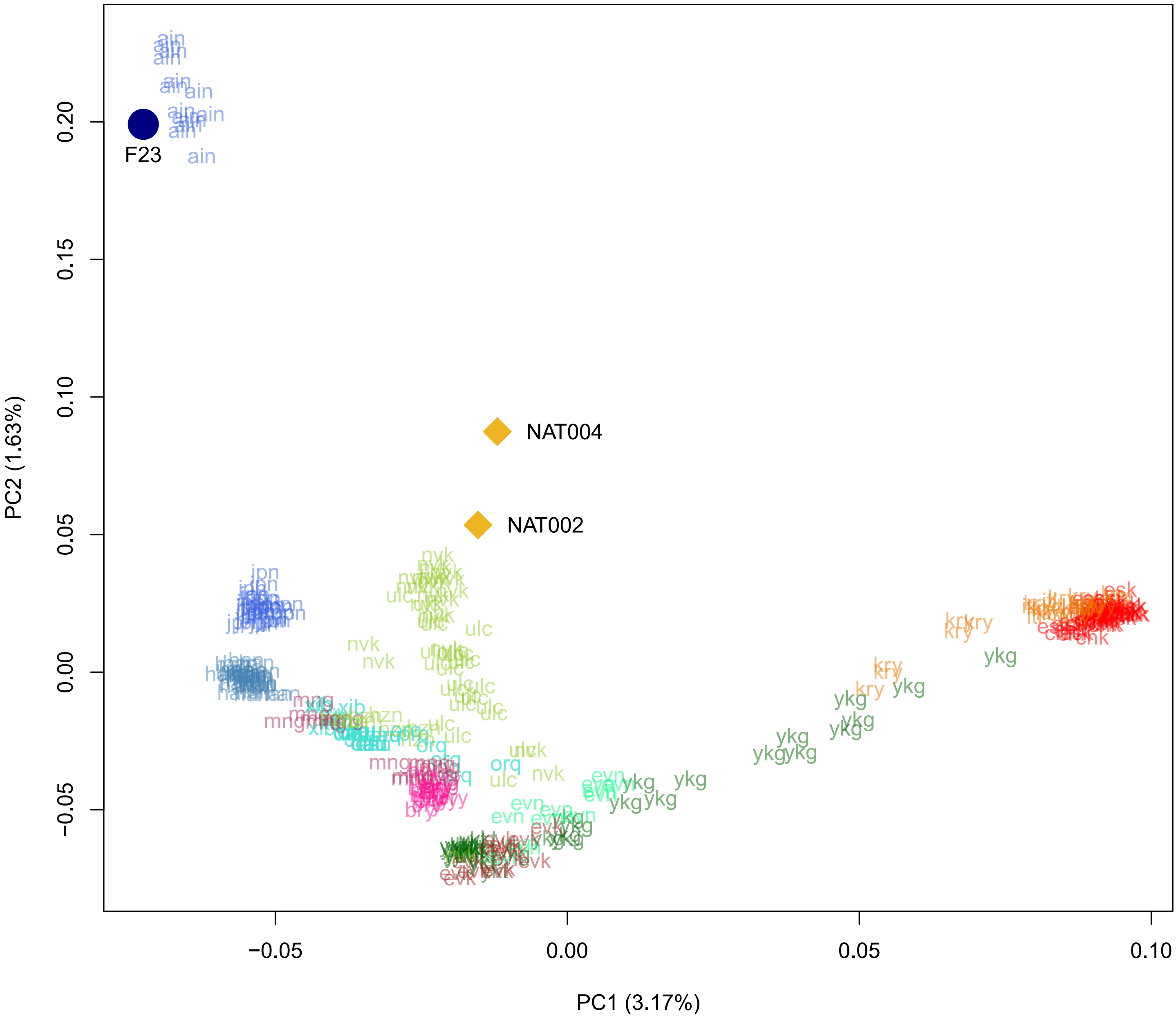


**Supplementary Figure S4** A PCA plot based on all SNV sites. Principal components were calculated using modern populations, and three ancient individuals (F23, NAT002, and NAT004) were projected onto the PC1-PC2 surface.

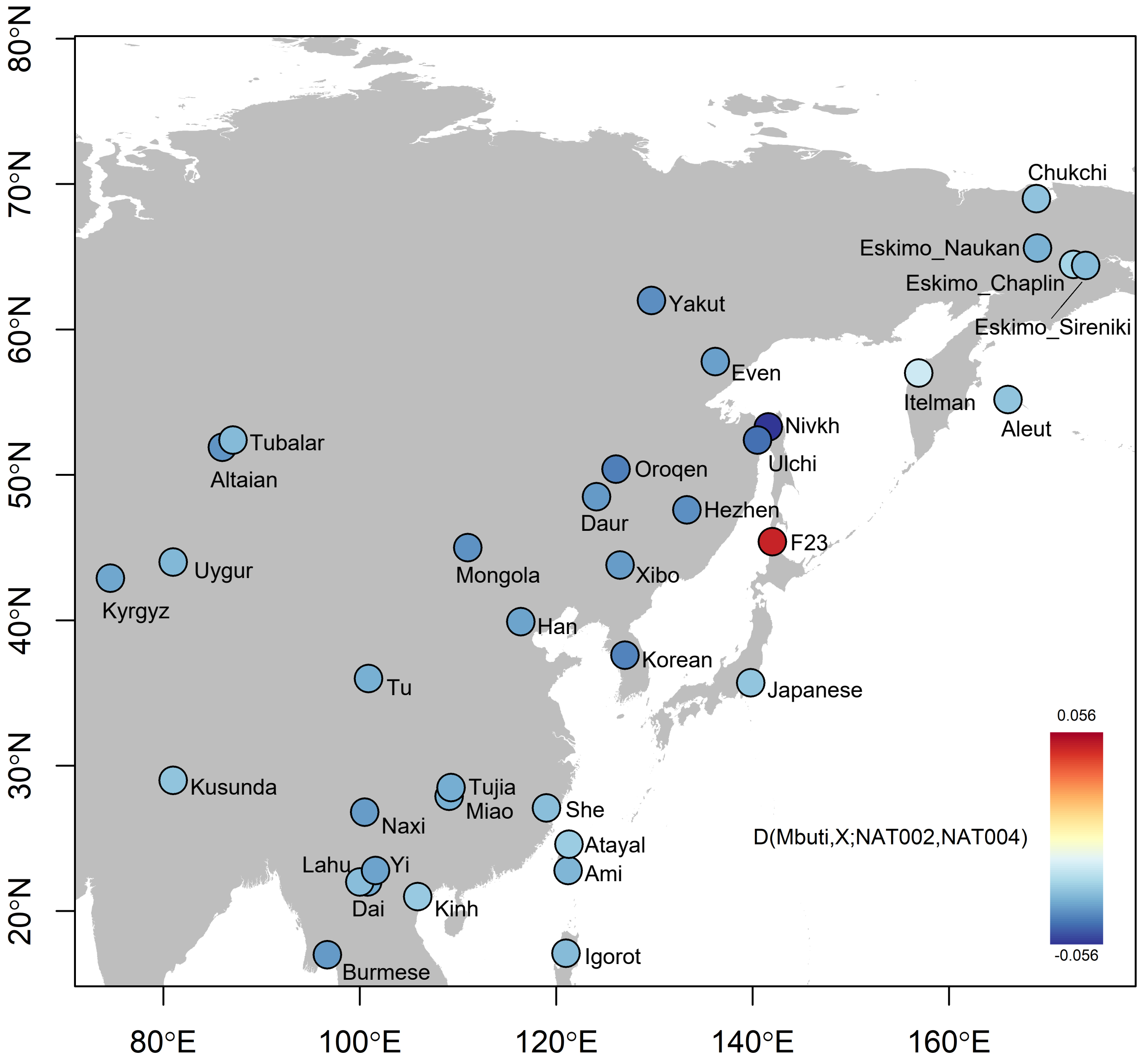


**Supplementary Figure S5** The result of *D* tests. *D*(Mbuti, X; NAT002, NAT004) was calculated. *D*-statistics for each population X except for the Itelman and F23 indicate the significant negative value, probably due to reference bias of low-coverage NAT004 genome.

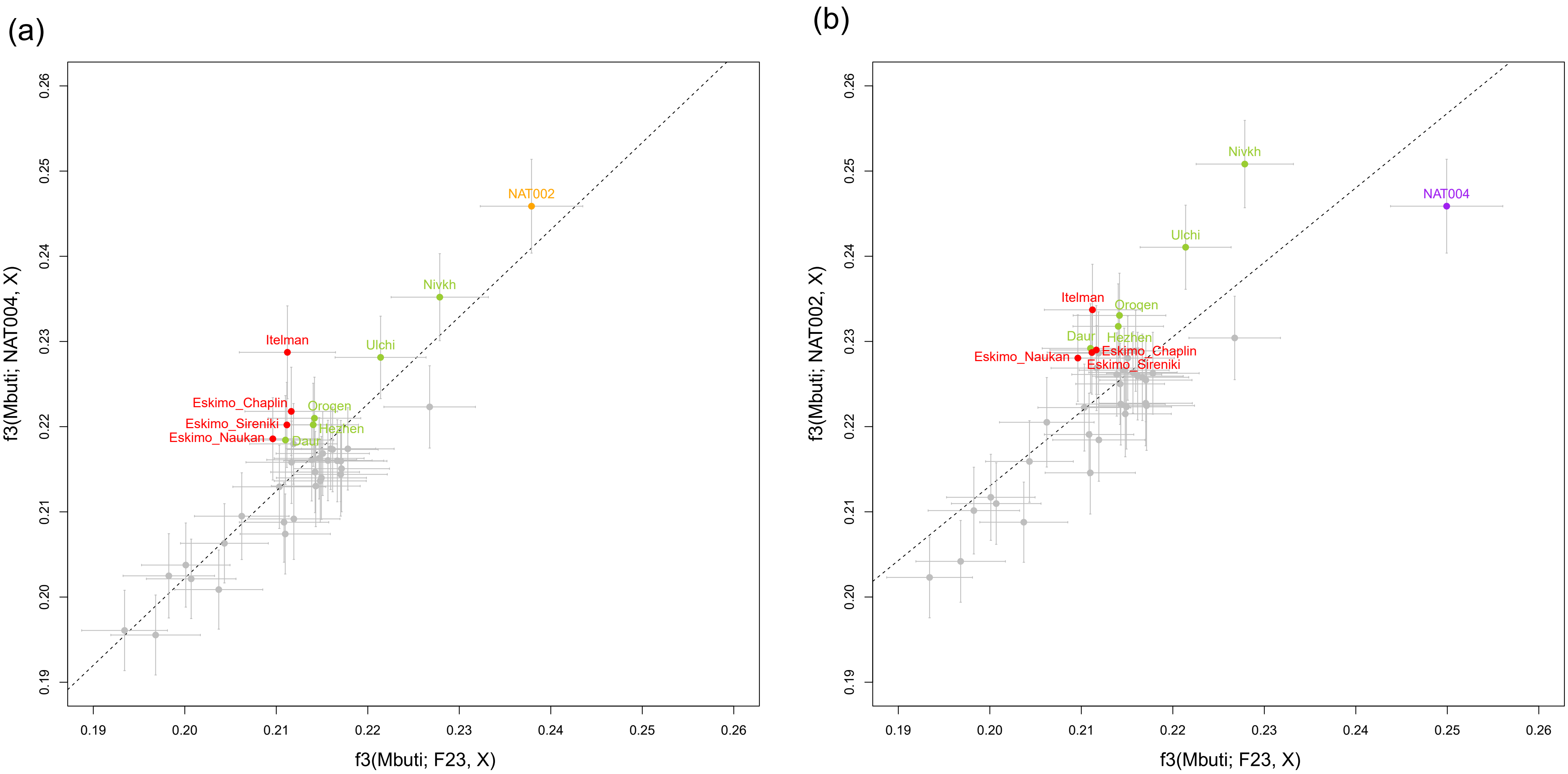


**Supplementary Figure S6** The pairwise $f_3$ plots. The dashed lines indicate the regression lines. Error bars indicate 2 standard errors. **(a)** $f_3$(Mbuti; F23, X) vs $f_3$(Mbuti; NAT004, X). Itelman, Eskimo_Chaplin, Eskimo_Naukan, and Eskimo_Sireniki significantly deviate from the regression line, suggesting that the population in northern Japan was genetically affected by the Kamchatka/Chukotka population from the late Jomon to early Okhotsk periods. **(b)** $f_3$(Mbuti; F23, X) vs $f_3$(Mbuti; NAT002, X). Addition to the Kamchatka/Chukotka populations, Nivkh, Ulchi, Oroqen, Hezhen, and Daur significantly deviate from the regression line, suggesting that the population in northern Japan was genetically affected by the Kamchatka/Chukotkapopulation and the Amur population from the late Jomon to late Okhotsk periods.

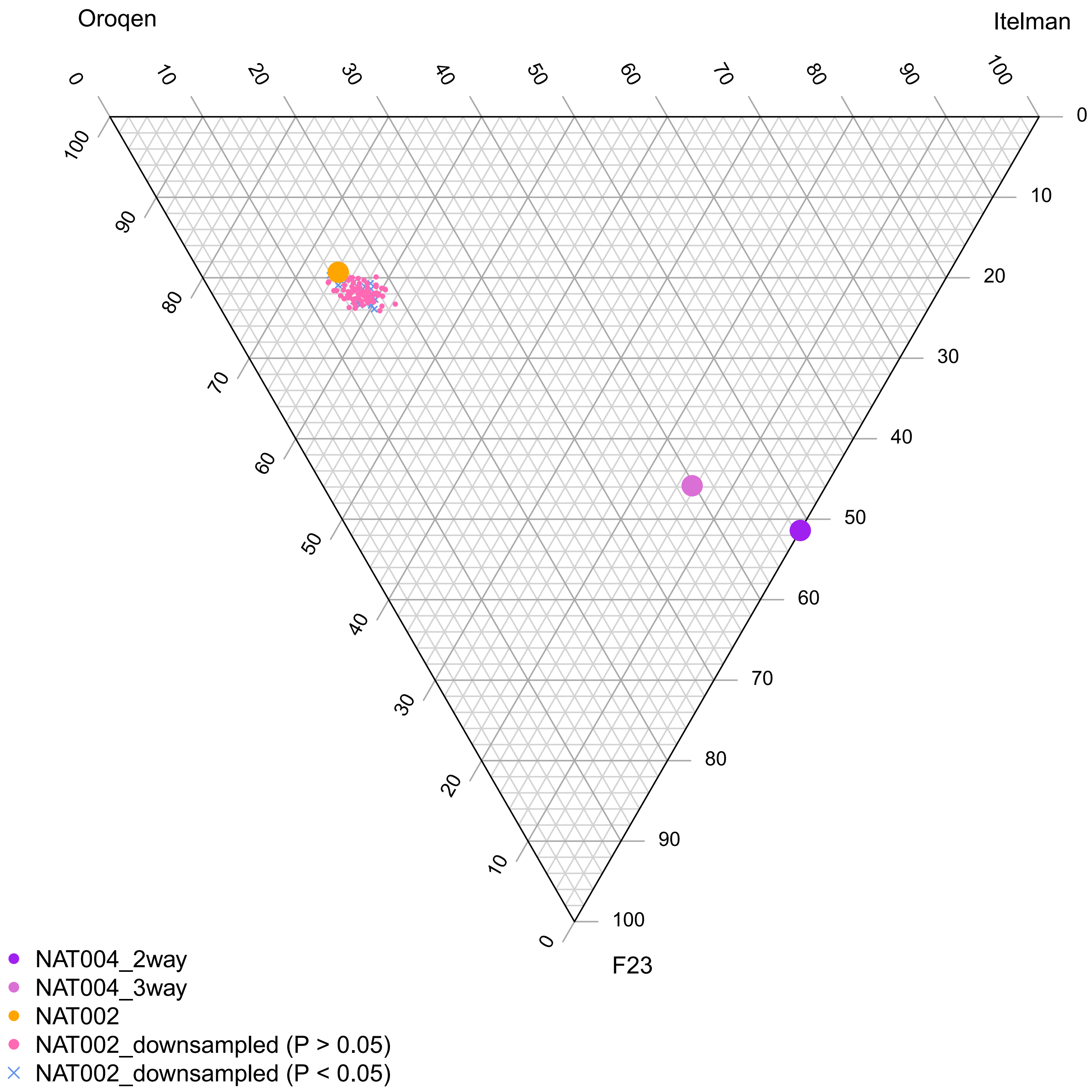


**Supplementary Figure S7** The ternary plot for qpAdm modeling. Although the down-sampled NAT002 data were slightly shifted, they were still plotted near the original position of NAT002. The result suggests that the lack or low-proportion of the Amur-related ancestry in NAT004 genome was not caused by the low-coverage of the obtained sequence data.

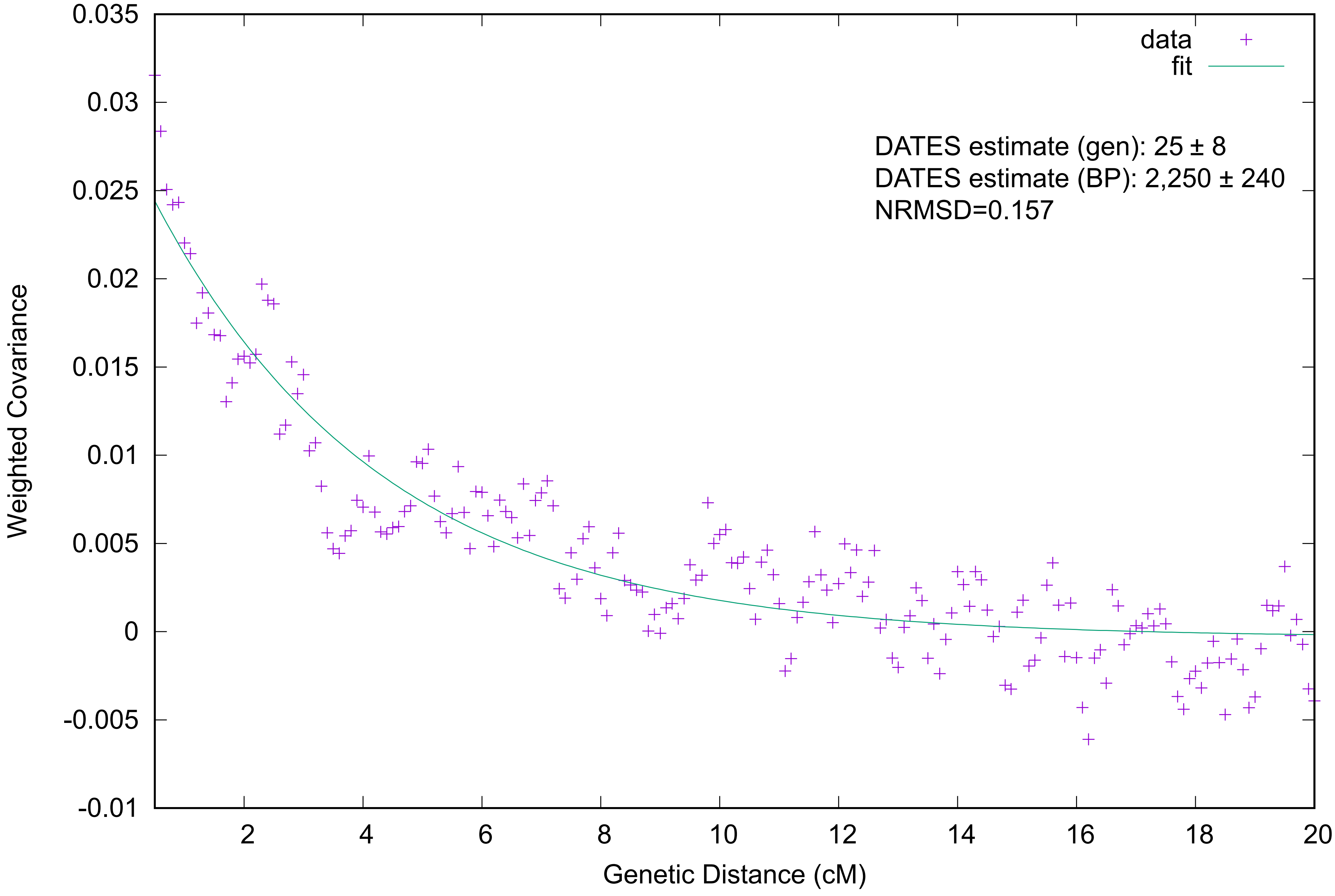


**Supplementary Figure S8** The inferred admixture date for NAT004, estimated using DATES by fitting a least-squares exponential model. F23 and the SGDP Itelman were used as source populations. DATES estimate (BP) was calculated assuming a generation time of 30 years and that NAT004 was a 1,500-year-old individual  based on the $^{14}C$ dating result.
